# *Caryocar brasiliense camb* protects against genomic and oxidative damage in urethane-induced lung carcinogenesis

**DOI:** 10.1590/1414-431X20154467

**Published:** 2015-07-21

**Authors:** N.B.R. Colombo, M.P. Rangel, V. Martins, M. Hage, D.P. Gelain, D.F. Barbeiro, C.K. Grisolia, E.R. Parra, V.L. Capelozzi

**Affiliations:** 1 Departamento de Patologia, Faculdade de Medicina, Universidade de São Paulo, São Paulo, SP, Brasil; 2 Laboratório de Poluição Atmosférica, Faculdade de Medicina, Universidade de São Paulo, São Paulo, SP, Brasil; 3 Departamento de Stress Oxidativo, Universidade do Rio Grande do Sul, Rio Grande do Sul, RS, Brasil; 4 Departamento de Emergência Clínica, Universidade de São Paulo, São Paulo, SP, Brasil; 5 Departamento de Genética e Morfologia, Universidade de Brasília, Brasília, DF, Brasil

**Keywords:** Pequi, *Caryocar brasiliense Camb*, Oxidative stress, Lung cancer, Urethane, Molecular biology

## Abstract

The antioxidant effects of *Caryocar brasiliense Camb*, commonly known as the pequi fruit, have not been evaluated to determine their protective effects against oxidative damage in lung carcinogenesis. In the present study, we evaluated the role of pequi fruit against urethane-induced DNA damage and oxidative stress in forty 8-12 week old male BALB/C mice. An *in vivo* comet assay was performed to assess DNA damage in lung tissues and changes in lipid peroxidation and redox cycle antioxidants were monitored for oxidative stress. Prior supplementation with pequi oil or its extract (15 µL, 60 days) significantly reduced urethane-induced oxidative stress. A protective effect against DNA damage was associated with the modulation of lipid peroxidation and low protein and gene expression of nitric oxide synthase. These findings suggest that the intake of pequi fruit might protect against *in vivo* genotoxicity and oxidative stress.

## Introduction

Lung cancer (LC) and other types of cancer are a major cause of death, and LC represents 28% of total deaths by cancer worldwide (1). The prevalence of LC has increased, whereas the mortality rate for neoplastic diseases has decreased in developed countries, in the last 20 years (1). Treatments include surgery, radiotherapy, and/or chemotherapy. However, chemotherapy has a number of limitations including side effects, toxicity, and drug resistance. Additionally, most established chemotherapy drugs lack specificity for tumor cells (2). Therefore, there has been a growing interest in the use of fruits as a promising source of more efficient new therapeutic anticancer drugs. Recent trends in cancer treatment also include increasing awareness and chemoprevention. These attitudes have opened the way to using natural or synthetic compounds to prevent the initiation and promotion of events associated with cancer development (1,3,4).

Epidemiological studies have highlighted an important relationship between increased consumption of antioxidant fruits and a lower risk of chronic diseases such as cancer (5). Therefore, the increased consumption of antioxidant fruits is considered a beneficial routine practice to decrease cancer incidence (3,4,). In this context, the antioxidant properties of fruits of the Caryocaraceae family have been previously investigated ([Bibr B10] [Bibr B11] [Bibr B12] 10-13). However, *Caryocar brasiliense Camb,* commonly known as pequi, a native fruit from the Brazilian savannah ([Bibr B10] [Bibr B11] [Bibr B12] 10-13) with antioxidant properties has not been assessed as a protective agent against oxidative damage in urethane-induced lung carcinogenesis.

Urethane metabolites cause oxidative stress in DNA molecules, causing the development of adducts and C-hydroxylation to form vinyl carbamate, which is then converted to an epoxide that interacts with nucleic acid (14). The resulting reactive oxygen species (ROS) and reactive nitrogen species (RNS) play an important role in the initiation, promotion, and progression phases of the disease (15,16). The endogenous antioxidant enzyme defense and adequate ingestion of antioxidants from exogenous sources prevent the oxidative damage caused by ROS and RNS, including DNA damage and lipid peroxidation (15).

In the present study, we evaluated *in vivo* DNA damage through biochemical, immunohistochemical, and molecular biology assays to assess the modulatory effects of pequi on the oxidative stress in urethane carcinogenesis.

## Material and Methods

### Plant material and chemicals

In the present study, plant material used as pequi oil and pequi ethanolic extract were provided by the laboratory of Departamento de Genética e Morfologia, Universidade de Brasilia, Brazil (PI0601631-6). The procedures used to obtain the pulp involved peeling or grating the internal mesocarp, which was stoked in a protected vessel and frozen at -86°C. Extraction of the pequi pulp was obtained by cold maceration using chloroform as a solvent. The extract was submitted to low-pressure evaporation and dried under high vacuum to remove the solvent completely. All the compounds were stored in dark flasks, labeled, and sealed to prevent oxidation until use. The relative composition of the pequi fruit pulp oil was previously determined by Miranda-Vilela et al. (12,13).

Xylazine, ketamine, and urethane were provided by the hospital pharmacy of the teaching hospital, Faculdade de Medicina, Universidade de São Paulo, Brazil. Beta-carotene (ß-carotene C9750) was obtained from Sigma Aldrich (USA). The corn oil used to dilute the beta-carotene was obtained from a local pharmacy.

### Urethane tumor induction

All procedures described in the project were approved by the local Ethics Committee for Animal Research (Internal Ethics Committee of the facilities, Faculdade de Medicina, Universidade de São Paulo, #020/11). Lung cancer was induced in mice with urethane (2× intra-peritoneal [*ip*] injections; 1.5 g·kg^-1^·dose^-1^), a chemical carcinogen (17). The use of urethane to induce this type of cancer is a simple and commonly used experimental model of lung cancer ([Bibr B18] [Bibr B19] 18-20).

### Animals and treatments

Male mice (BALB/C, n=40, 8-12 weeks old) were obtained from the animal facility of the Faculdade de Medicina, Universidade de São Paulo. The animals were housed in plastic cages (6 per cage) at room temperature and were provided free access to food and water. On the same day as the second administration of urethane, the animals were divided into 5 groups and started to receive antioxidant supplementation. The control group (C, n=5) did not receive gavage or urethane doses. All other groups received two doses of urethane and specific gavage treatment as follows. The urethane-injected group (U, n=5) received 2 doses of urethane and did not receive gavage. The pequi ethanolic extract group (UE, n=10) received urethane (2 doses) and daily gavage with ethanolic extract (15 µL) of pequi. The pequi oil group (UO, n=10) received urethane (2 doses) and 30 mg·animal^-1^·day^-1^ of pequi oil administered orally as 15 µL by gavage. The beta-carotene group (UB, n=10), received urethane (2 doses) and daily gavage with beta-carotene (3 µg/kg, diluted in corn oil). After treatment (60 days), the animals received an *ip* dose of a lethal mixture of ketamine and xylazine. The lungs were then removed *en bloc*. To avoid regional differences in lung specimens, we randomized tissue samples including most of the lung lobes for the different assays.

The optimal doses of pequi oil and pequi extract used in this study were established by conducting two pilot studies as well as previously published data ([Bibr B11] [Bibr B12] 11-13). The dose of beta-carotene administered was calculated by transforming the equation developed by Reagan-Shaw et al. (20), and using a dose close to that of the nutritional recommendations by the Brazilian Agency for Sanitary Surveillance for provitamin A carotenoids.

### Determination of antioxidant enzyme activity in lung tissue

Lung tissue catalase (CAT) activity was assessed by measuring the rate of decrease in H_2_O_2_ absorbance using a spectrophotometer (240 nm). The results are reported as CAT units/mg protein. Superoxide dismutase (SOD) activity was assayed by quantifying the inhibition of superoxide-dependent adrenaline auto-oxidation using a spectrophotometer (480 nm) and the results were reported as SOD units/mg protein. The ratio between SOD and CAT activities (SOD/CAT) was calculated to analyze the effect of treatments with pequi oil and ethanolic extract. Lung tissue glutathione peroxidase (GPx) activity was determined by measuring the rate of NADPH oxidation using a spectrophotometer (340 nm). The activity of GPx was reported as International Units (nmol NADPH oxidized·min^-1^·mg protein^-1^.

### Comet assay

Fresh lung tissue was macerated and homogenized. Cells embedded in agarose on a microscope slide were lysed with detergent and high salt to form nucleoids containing supercoiled loops of DNA linked to the nuclear matrix. Electrophoresis at pH 7 resulting in structures resembling comets was performed as previously described (21,22). Damaged cells were identified by the presence of a tail similar to that of a comet, formed by DNA fragments. The fragments were present in different sizes and were associated with the damage score.

### Thiobarbituric acid reactive substances (TBARS) assay

To evaluate lipid peroxidation, the formation of TBARS was measured during an acid-heating reaction, which is commonly used for the measurement of the lipid redox state. The samples were mixed with trichloroacetic acid (10%, 0.6 mL) and thiobarbituric acid (0.67%, 0.5 mL), and then incubated at 100°C for 25 min. TBARS were determined by their absorbance measured using a spectrophotometer (532 nm). The results are reported as nmol TBARS/mg protein.

### Immunohistochemistry

Immunohistochemistry to detect the expression of the NOS1, NOS2, and NOS3 isoforms (Santa Cruz Biotechnology, Inc., USA; dil. 1:100) was performed using a standard peroxidase technique. Samples were diluted in a bovine serum albumin (BSA; 0.5%, Sigma Diagnostics, USA) solution. The antigen expression was determined using Novolink Max Polymer (Leica Biosystems Newcastle Ltd., UK), pressure-cooking antigen retrieval, diaminobenzidine tetrahydrochloride, and counterstaining with hematoxylin. Brownish cytoplasmic staining in the alveolar and bronchiolar cells was considered evidence of antigen expression.

Expression of NOS1, NOS2, and NOS3 was assessed in 10 fields using a point-counting technique with a 100-point grid of a known area (62,500 mm^2^; 400× magnification) attached to the microscope ocular (23). The area of each field was calculated according to the number of points containing positive cells for NOS1, 2, and 3, proportional to the total grid area (400× magnification). The fractional area of positive cells was determined as the number of positive cells in each field divided by the connective tissue area. The final results are reported as percentages.

Interobserver comparisons were performed in 20% of the slides by two observers (VLC and ERP). The coefficient of variation for interobserver error regarding cell count was <5%.

### Molecular biology

Total RNA was extracted using the Trizol method as described previously (24). Complementary DNA (cDNA) was synthesized using total RNA (4 µg) and the SuperScript First-Strand Synthesis System for RT-PCR Kit (Invitrogen life Technology, UK). Quantitative RT-PCR was performed using the TaqMan assay according to the manufacturer's instructions for gene expression quantification of NOS1, NOS2, and NOS3. Reactions were performed on the StepOnePlus (Applied Biosystems, USA) system. Data were analyzed by StepOne (v. 2.0; Applied Biosystems) software. Results displaying a CT intra-variation <1.5 were further used to calculate mean values. Data are reported as CT values (the cycle number at which logarithmic PCR plots cross a calculated threshold line). The relative expression of genes of interest was normalized to that of GAPDH and gene expression in each sample was then compared with the expression in pool cells. The comparative CT method was used to quantify gene expression and the relative expression was calculated as 2 CT.

### Statistical analysis

All data were analyzed using statistical tests for differences, based on the distribution types of variables. Parametric distributions were performed using the SPSS (Statistical Package for Social Sciences; v. 18.0; SPSS Inc., USA) statistical software. Values of each continuous variable are reported as means±SD. The Student's *t*-test was used to compare the means of two groups of samples. Mean comparison of three or more groups of samples was performed by the *t-*test and one-way analysis of variance (ANOVA) followed by appropriate *post hoc* tests, including the Bonferroni test for multiple comparisons. P<0.05 was considered to be statistically significant.

## Results

### Urethane tumor induction

**Figure 1:**
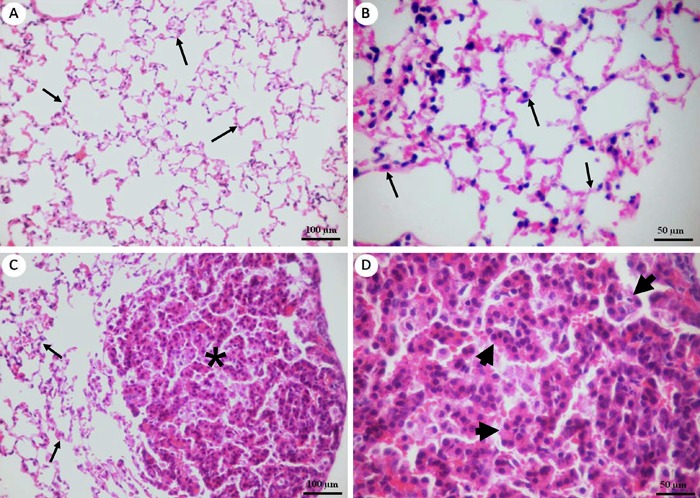
Histopathology of lung tissue. *A* and *B*, Preserved alveolar acini with delicate alveolar walls (arrows) in control mice (H&E). *C* and *D*, Lung tissue of urethane-injected mice showing atypical epithelial nodules (asterisk) with subpleural localization composed of round or oval cells (arrowheads) containing abundant eosinophilic cytoplasm and hyperchromatic nuclei, but without evident nucleoli, forming characteristic glands or acini (H&E).

Lung tissue specimens from control animals were analyzed at a low magnification and showed uniform histoarchitecture of the lung parenchyma (Figure 1A) and thin alveolar walls (Figure 1B). In contrast, lung specimens from urethane-injected mice were modified by the presence of small subpleural nodules in the parenchyma (Figure 1C and D) including slightly atypical round or oval epithelial cells, containing hyperchromatic nuclei and eosinophilic cytoplasm arranged in small acini. The atypical mitosis count was in the range of 3-5 per 10 high-power fields.

### Determination of antioxidant enzyme activity in lung tissue

**Figure 2:**
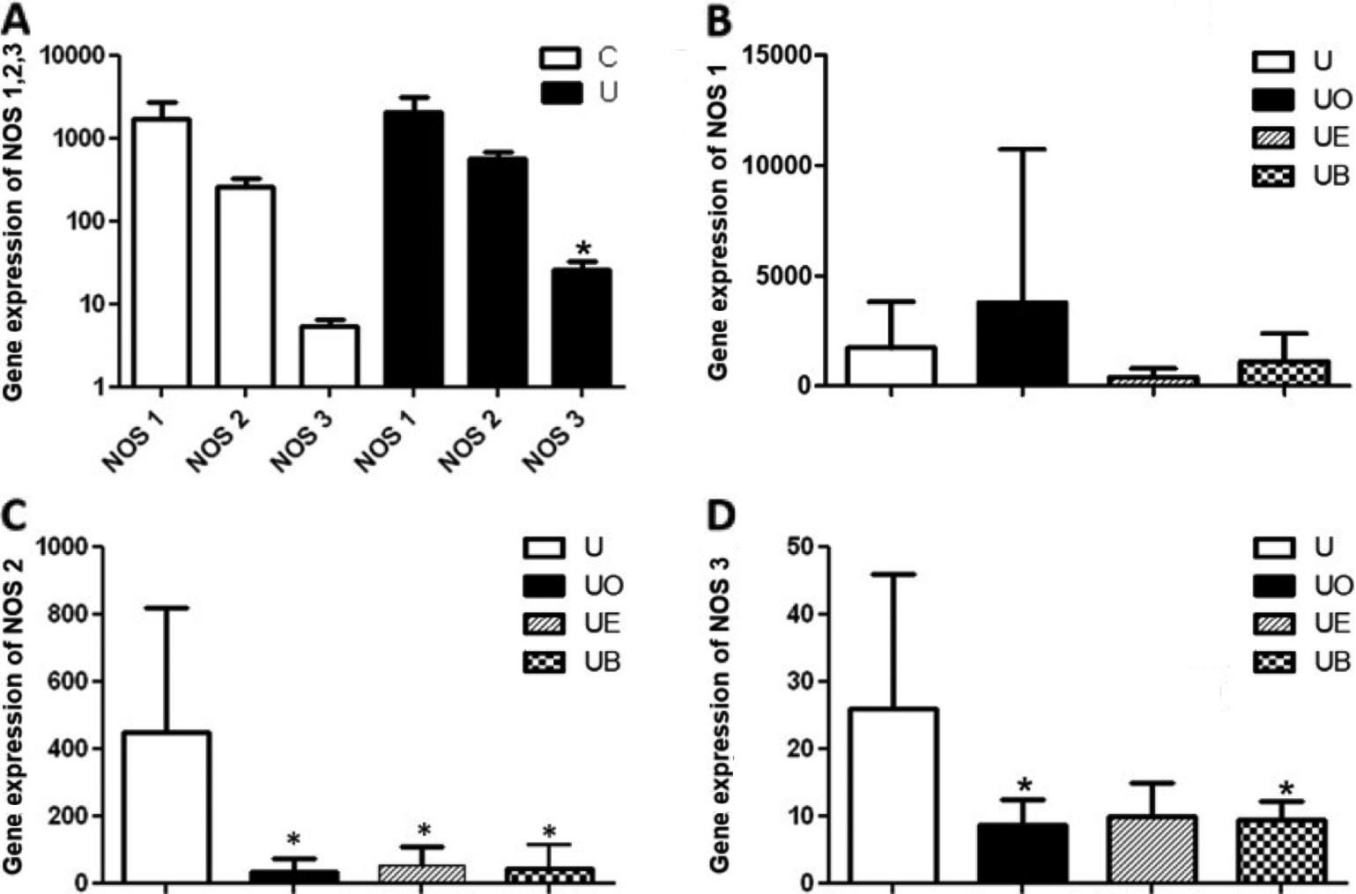
Quantification of activity of antioxidant enzymes in lung tissue specimens. *A*, Urethane-injected mice (U) had higher catalase (CAT) (*P=0.003) and superoxide dismutase (SOD) (P=0.07) activities compared to controls (C). *B*-*E*, There were no significant differences between the activities of CAT (*B*), SOD (*C*), SOD/CAT ratio (*D*), and glutathione peroxidase (CPx) (*E*). UO: urethane + pequi oil; UE: urethane + pequi ethanolic extract; UB: urethane + beta-carotene. ANOVA was used for statistical analysis.

Catalase, superoxide dismutase, and glutathione peroxidase levels were analyzed in lung tissue specimens from control and urethane-injected mice. The SOD/CAT ratio was also determined. In lung tissue specimens from urethane-injected mice, although showing a borderline significance (P=0.05), we observed a higher catalase activity than in control mice (Figure 2A). In contrast, SOD and glutathione peroxidase activities were similar in both groups. Similar antioxidant enzyme activity was observed in lung tissue from supplemented and urethane-injected mice (Figure 2B-D).

### Thiobarbituric acid reactive substances (TBARS) assay

**Figure 3:**
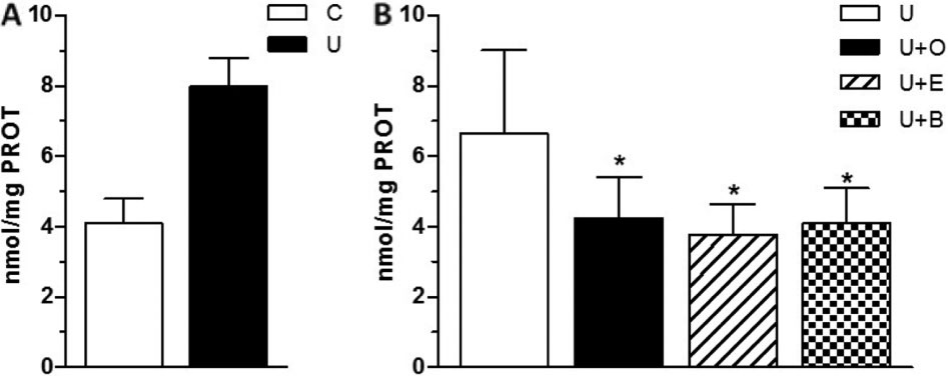
Lipid peroxidation in lung specimens of mice. *A*, In urethane-injected mice (U), lipid peroxidation was higher than in control mice (C) (P=0.05). *B*, In animals that received daily doses of antioxidants, the degree of lipid oxidation was lower than that observed in urethane-injected mice (P<0.05). Data are reported as means±SD for urethane-injected and control mice (n=5) and urethane-injected and supplemented mice (n=10). U+O: urethane + pequi oil; U+E: urethane + pequi ethanolic extract; U+B: urethane + beta-carotene. *P<0.05, ANOVA and *t*-test.

Figure 3 shows lipidic peroxidation in lung specimens of urethane-injected and supplemented mice. In lung specimens of urethane-injected mice, oxidative damage to polyunsaturated fatty acids of cell membranes was higher than in those of control mice (Figure 3A). In lung specimens of animals supplemented with pequi oil and extract and beta-carotene, the levels of lipid peroxidation were lower than in those of urethane-injected mice (Figure 3B).

### Comet assay

**Figure 4:**
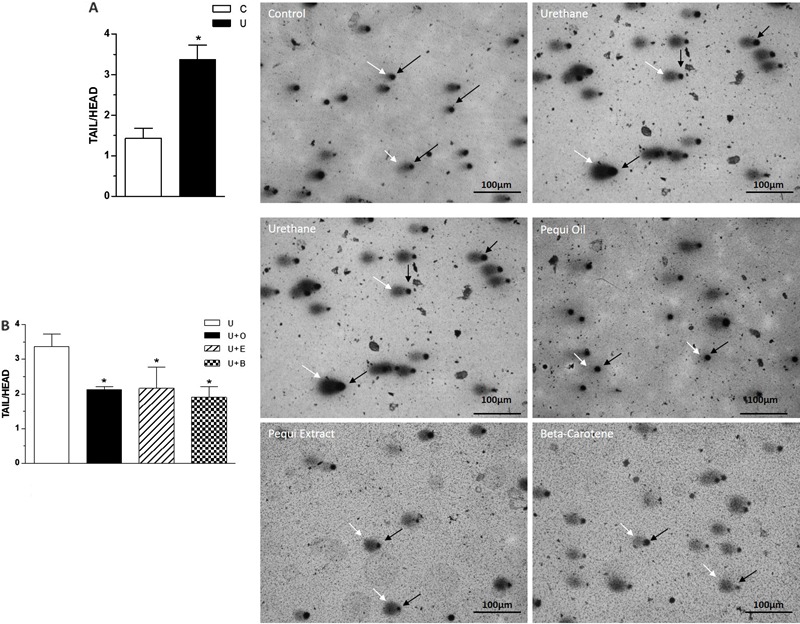
Oxidative damage to DNA in lung tissue specimens. *A*, Lung tissues from urethane-injected mice (U) showed higher DNA damage when compared to control mice (*P<0.0001, *t*-test). The degree of DNA damage is illustrated in lung cells of control mice and urethane-injected mice (arrows). *B*, The ratios of tail/head as measured in the comet assay of supplemented mice (U+O: pequi oil group; U+E: pequi ethanolic extract group; U+B: beta-carotene group) show reduced DNA damage compared to urethane-injected mice (*P<0.0001, ANOVA; arrows). Data are reported as means±SD in urethane-injected and control mice (n=5) and urethane-injected and supplemented mice (n=10). Black arrows show the head and white arrows show the tail.

Distribution of comet tail/head ratio in urethane-injected and urethane-supplemented groups is shown in Table 1. In lung tissue specimens, oxidative damage to DNA was observed in the urethane-injected mice and was stratified according to the groups (Figure 4). Lung tissue from urethane-injected mice showed a higher degree of DNA damage (Figure 4A), which was compatible with the increased comet tail/head ratio, when compared with control mice. Interestingly, lungs from animals that received the antioxidant supplementation showed a lower degree of DNA damage (Figure 4B).

**Table:**
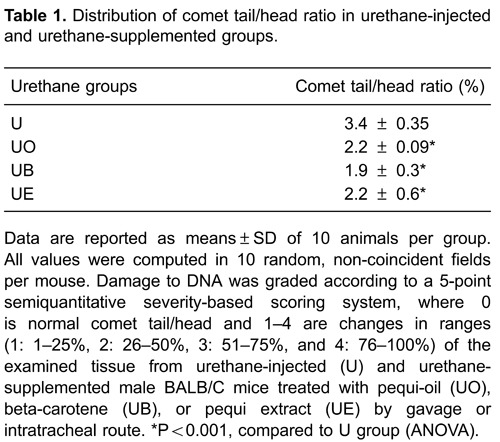

### Immunohistochemistry

**Figure 5:**
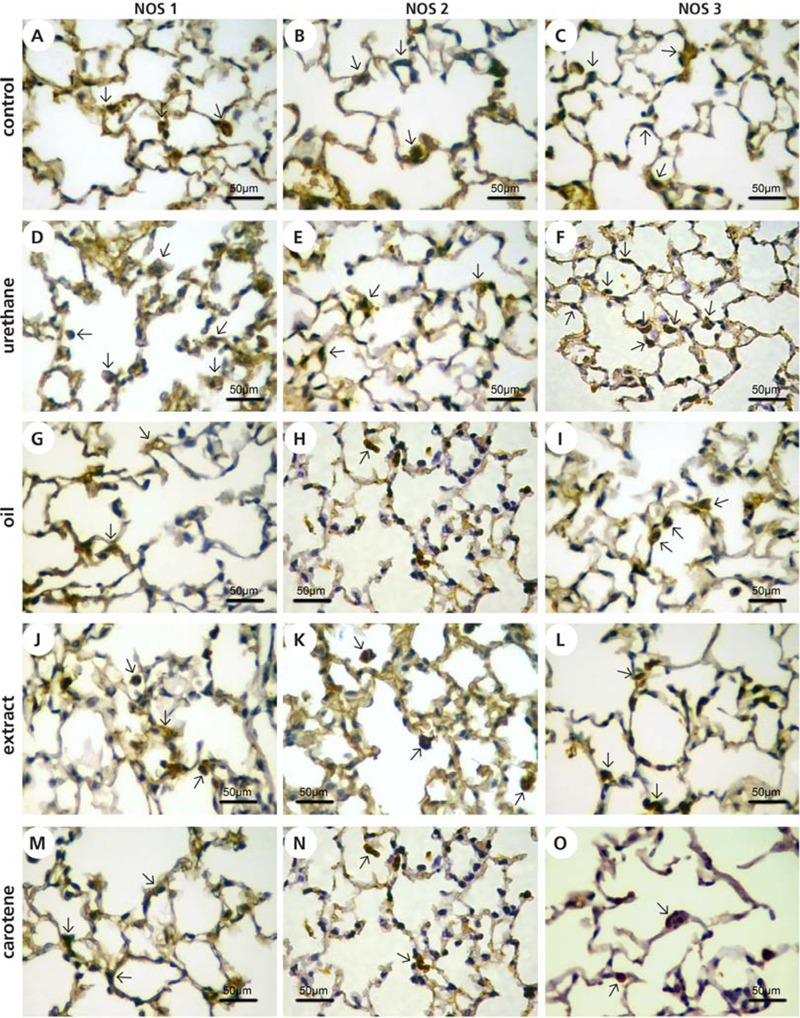
Immunostaining for NOS1, 2, and 3. Representative NOS-stained sections of lung tissue specimens from control, urethane-injected, and supplemented mice are shown. *A*-*C*, Few alveolar epithelial cells (arrows) in control mice express NOS1, 2, or 3. *D*-*F*, Numerous alveolar epithelial cells (arrows) in urethane-injected mice express NOS1, 2, or 3. *G*-*O*, Alveolar epithelial cells (arrows) in antioxidant-supplemented mice with reduced expression of NOS1, 2, and 3.

**Figure 6:**
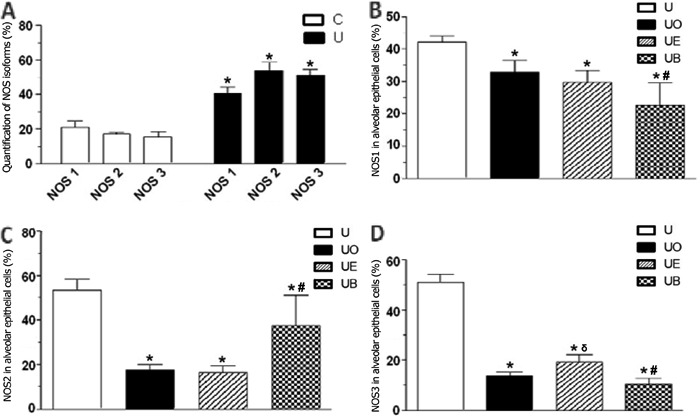
Quantification of NOS immunoexpression in lung tissue specimens. *A*, NOS in urethane-injected (U) and control mice (C), *P<0.0001. *B*, NOS1 in urethane-injected and supplemented mice: *pequi oil group (UO), pequi ethanolic extract group (UE), and beta-carotene group (UB) *vs* urethane-injected group (U) (P<0.001), and ^#^UB *vs* UE and UO (P<0.05). *C*, NOS2 urethane-injected and supplemented mice: *U *vs* UO, UE, and UB (P<0.0001), and ^#^UB *vs* UO and UE (P<0.05). *D*, NOS3 in urethane-injected and supplemented mice: *U vs UO, UB, and UE (P<0.001), ^#^UB *vs* UO and UE (P<0.001), and ^δ^UE *vs* UO (P<0.001). Data are reported as means±SD in urethane-injected and control mice (n=5) and urethane-injected and supplemented mice (n=10). Groups were compared using ANOVA and the *t*-test.

Different immunostaining intensities were observed in the alveolar cells of lung tissue from urethane-injected and supplemented mice (Figure 5), and were confirmed by histomorphometric analysis (Figure 6).

The immunoexpression of NOS1, 2, and 3 in lung alveolar cells of urethane-injected mice was higher than that observed in control animals (Figure 6A). In contrast, lungs from mice that received antioxidant supplementation exhibited a decreased immunoexpression of NOS1, 2, and 3 when compared to the urethane-injected group (Figure 6B, C, and D). Interestingly, an inverse order was observed for NOS expression in mice supplemented with beta-carotene. The lung expression of NOS1 (Figure 6B) and NOS3 (Figure 6D) was lower and that of NOS2 (Figure 6C) was higher than in mice supplemented with pequi oil and pequi extract. In addition, NOS3 expression in mice supplemented with pequi oil was lower than in mice supplemented with pequi extract (Figure 6D).

### Gene expression of nitric oxide synthases

**Figure 7:**
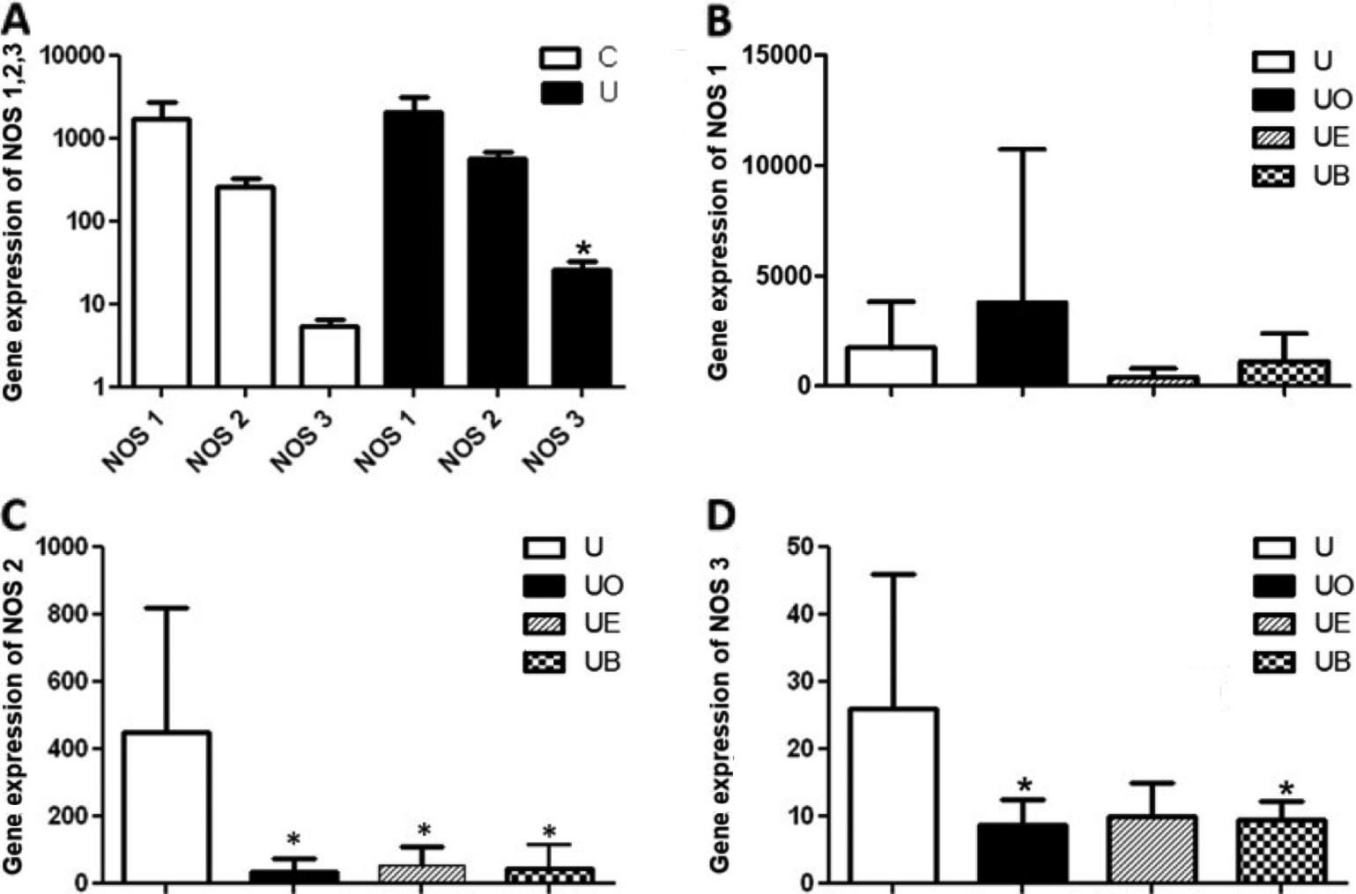
Gene expression of nitric oxide synthases. *A*, Expression of NOS1, 2, 3 in urethane-injected mice (U) was significantly higher than in control mice (C) (*P<0.05). *B*, There was no significant difference in the gene expression of NOS1 in the study groups. *C*, Expression of NOS2 in urethane-injected mice was higher than in antioxidant supplemented mice, *P<0.05, pequi oil group (UO), pequi ethanolic extract group (UE), and beta-carotene group (UB) *vs* urethane-injected group (U). *D*, Expression of NOS3 in mice supplemented with pequi oil and beta-carotene was lower than in urethane-injected mice, *P<0.05, U *vs* UO and UB. Data are reported as means±SD in urethane-injected and control mice (n=5) and urethane-injected and supplemented mice (n=10). Groups were compared using the ANOVA and the *t-*test.

The gene expression of *NOS* for different groups was analyzed (Figure 7). In lung tissue specimens from urethane-injected mice, the gene expression of *NOS3* was higher than in the controls. Figure 7B shows that the supplementation of urethane-injected mice with pequi oil or extract did not modify the gene expression of *NOS1* in lung tissues. The gene expression of *NOS2* in lung tissues from supplemented mice was lower than in urethane-injected mice (Figure 7C). In mice supplemented with pequi oil and beta-carotene, *NOS3* gene expression was lower than in urethane-injected mice (Figure 7D).

## Discussion

In this study, we analyzed the modulatory effects of pequi fruit on oxidative stress induced by urethane carcinogenesis by measuring *in vivo* DNA damage through biochemical, immunohistochemical, and molecular biology assays. Beta-carotene was used as a positive control. We showed that the popular pequi fruit has anti-DNA damage and antioxidant properties. Specifically, we showed a significant protective effect of pequi fruit oil and pequi extract against DNA and oxidative damage induced by urethane in the lung. Our findings using biochemical and molecular biology assays indicated that the antioxidant activity of *Caryocar brasiliense Camb* might be useful as an antioxidant to reduce the effects of genotoxic carcinogens.

After 60 days of treatment, urethane-injected mice developed small subpleural tumors in the lung parenchyma. Urethane tumors are a neoplasm of epithelial malignant origin corresponding to the human adenocarcinoma (25). Urethane cancer is a rapid model of lung carcinogenesis because nodules are formed within 2 months (18). The process of urethane carcinogenesis involves enzyme metabolization into vinyl carbamate and N-hydroxylamine epoxides, which generate oxidative stress in the lung cell environment by generating ROS and NOS (15), which cause oxidation and DNA damage (16). In the nitrogen cycle, nitrogen oxide is critical in converting nitrate (NO_3_^-^) and nitrite (NO_2_^-^) to ammonia (NH_4_^+^), an essential component of protein synthesis, as well as in forming vascular tone and cell signaling regulation. However, nitrogen oxide is also associated with the generation of carcinogenic nitrosamines (26). In this circumstance, NO might contribute to the induction of genotoxic lesions as well as the promotion of angiogenesis, tumor cell growth, and invasion (27). In mammals, NO is synthesized by three differently gene-encoded NOS: neuronal NOS (nNOS or NOS1), inducible NOS (iNOS or NOS2), and endothelial NOS (eNOS or NOS3). The three isoforms share similar structures and catalytic characteristics, although the mechanisms that control their activity in time and space are different (28). The expression of NOS2 is induced by an inflammatory signal, whereas NOS1 and NOS3 are constitutively expressed (29).

Our results confirm the reports cited above. Indeed, these three NOS isoforms were highly expressed in alveolar cells in the lung tissue of urethane-injected mice. Moreover, a high gene expression of *NOS2* and *NOS3* was found in lung tissue specimens from urethane-injected mice. Nevertheless, lungs from animals supplemented with pequi had a decreased expression of these isoforms, and *NOS3* was diminished further after supplementation with pequi oil when compared with pequi extract. These findings are in accordance with the lower gene expression of *NOS2* and *NOS3* in lung tissues from mice supplemented with pequi oil and beta-carotene, without modification of *NOS1* gene expression. Therefore, these findings might be related to the carotenoid component found in the pequi oil and extract (30), and also to its antioxidant activity reinforced by the presence of the DPPH (2,2'-diphenyl-1-picrylhydrazyl) free radical (31). However, although the carotenoid content might be partly responsible for the protective effect of pequi oil as observed in our study, other components of pequi oil such as oleic, palmitic, stearic, and polyunsaturated fatty acids might also have some effects. The antineoplastic effect of oleic acid was previously described (32), and palmitic, stearic (saturated), and linolenic (polyunsaturated) fatty acids are related to prostate cancer (33) and dietary polyunsaturated fatty acids with breast and colorectum tumors (34).

Interestingly, we found an inverse association between NOS protein expression and mice supplemented with beta-carotene. In these mice, the lung expression of the NOS1 and NOS3 isoforms was lower and NOS2 expression was higher than in mice supplemented with pequi oil or pequi extract. A pro-oxidant effect, characterized by an increased incidence of lung cancer, was described in smokers taking high (pharmacological) daily doses of beta-carotene for 6 months (35). It was also suggested that pro-oxidant compounds increase the production of free radical species, neutralizing antioxidant defenses and causing damage to cell membranes, proteins, and DNA (36). Taken together, these findings support the concept that antioxidant supplementation exerts different actions on the different NOS isoform proteins. For example, the administration of beta-carotene increased the expression of the NOS2 isoform, which could be detrimental in a model of tumor-bearing mice. Supplementation with the pequi extract and pequi oil reduced the expression of the three NOS isoform proteins, but only reduced the gene expression of the *NOS2* and *NOS3* isoforms. The gene expression of *NOS1* was unchanged with supplementation.

We also found that the oxidative damage to polyunsaturated fatty acids in the cell membranes of lung tissue from urethane-injected mice was higher than in control mice. This finding is in agreement with the evidence that excess free radicals generated by urethane causes oxidative damage to the cellular components of lung parenchyma, including DNA. This damage leads to the constitutive activation of signaling pathways, promoting and initiating a cascade of lipid peroxidation and protein oxidation that elicits inflammation-driven carcinogenesis (37). Our study used the comet and oxidative stress assays to investigate the cytotoxic, genotoxic, and oxidative effects of urethane involved in carcinogenesis-bearing mice. Significant cytotoxic and genotoxic effects were observed after urethane injection, which induced a significant reduction in cell viability with clear direct DNA damage mediated by oxidative stress. Such effects were inhibited after the supplementation of mice with pequi and beta-carotene. Therefore, DNA damage can be avoided through antioxidant status modulation by administering pequi oil or pequi extract, resulting in protection against the genotoxic effects of urethane.

A high activity of catalase, superoxide dismutase, and glutathione peroxidase was maintained in the lung tissue of urethane-injected mice after supplementation with pequi oil or pequi extract, and might be explained as an initial response to the oxidative damage, because the cellular oxidative status regulates the activity of these enzymes (38). We also concluded this was a compensatory mechanism to prevent oxidative damage. Nevertheless, this result may also be interpreted as the insufficient inhibition or decrease of oxidative stress caused by urethane, suggesting that increased ROS production and reduced ROS detoxification are involved with tumor growth and metastasis as previously reported (39). Our results indicated that continuously administered pequi oil enhanced the activity of antioxidant enzymes such as glutathione reductase, glutathione S-transferase, catalase, and superoxide dismutase in urethane primed cancerous lung tissues of mice (40).

To the best of our knowledge, the present study is the first to investigate the effects of pequi fruit pulp oil (a carotenoid-rich oil) and extract on experimental lung cancer, the expression of antioxidant enzymes, protein and gene expression, as well as DNA conformational changes in cancerous cells. However, our study limitations included the comparison of results. Other studies on the effects of pequi fruit on tumorigenesis conducted by the Miranda-Vilela group (12,13) used Ehrlich tumor-bearing mice to investigate hematological, toxicological, and histopathological changes.

Based on the results and observations, our study indicated that pequi oil and pequi extract modified urethane lung cancer in BALB/C mice due to its antioxidant properties, and restored urethane-mediated conformational changes of DNA to normal. We postulated that pequi may improve the antioxidant defense system, by increasing the activities and expression of antioxidant enzymes at the protein and genomic level, thus reducing oxidative stress, consequently inhibiting the overexpression of transcription factors. Our results also suggested that pequi may modify the carcinogenic process either by blocking the development of early lesions or by inhibiting the progression to invasive cancer. Further investigation is required to elucidate the role of each active constituent of pequi to determine the molecular mechanism involved and to develop targeted therapy for lung and other cancers.
